# Diagnosis and Management of a Patient With 5-Fluorouracil-Induced ST Elevation and Nonsustained Ventricular Tachycardia as a Late Presentation of Cardiotoxicity and Successful 5-Fluorouracil Rechallenge

**DOI:** 10.7759/cureus.30489

**Published:** 2022-10-19

**Authors:** Lalitha C Medepalli, Tariq S Mahmood, Henry Liberman, Anita M Medepalli, Thomas W Bagwell

**Affiliations:** 1 Cardiology/Cardiooncology, Northside Hospital Atlanta/Northside Hospital Cardiovascular Institute, Atlanta, USA; 2 Medical Oncology, Northside Hospital Atlanta/Atlanta Cancer Care, Atlanta, USA; 3 Interventional Cardiology, Northside Hospital Atlanta/Northside Hospital Cardiovascular Institute, Atlanta, USA; 4 Medicine, Mercer University School of Medicine, Macon, USA

**Keywords:** cardio-oncology, vasodilator therapy, rechallenge of 5-fu, st elevation, chest discomfort, colorectal cancer, 5-fu

## Abstract

5-fluorouracil (5-FU) is an antimetabolite drug that is used in the treatment of a variety of carcinomas, including breast, gastric, pancreatic, colon, and rectal cancers. It is usually administered to decelerate and prohibit cancer cell proliferation. It acts by inhibiting the enzyme thymidylate synthase by blocking thymidine formation required for deoxyribonucleic acid (DNA) synthesis. The most common clinical manifestation of 5-FU cardiotoxicity is chest pain related to coronary vasospasm. Patients experiencing cardiotoxicity induced by 5-FU present with signs and symptoms of acute coronary syndromes with elevated cardiac biomarkers (troponin), and their ECGs often reveal ST segment changes. There can be two distinct clinical presentations, early or late presentation of cardiotoxicity. Early toxicity can occur during the infusion, whereas late presentation of toxicity can occur 1-2 days after the infusion. Usually, with early toxicity, troponin elevation may be evident. However, in late presentation of cardiotoxicity symptoms, troponin elevation and/or ECG changes may be undetectable. Our case has a unique presentation of 5-FU toxicity in a patient developing ST elevation and non-sustained ventricular tachycardia (VT) as a late presentation of cardiotoxicity.

## Introduction

5-fluorouracil (5-FU) is an antimetabolite drug that is used to treat cancer. It is usually administered to decelerate and prohibit cancer cell proliferation. The most common clinical manifestation of 5-FU cardiotoxicity is chest pain related to coronary vasospasm. Patients experiencing cardiotoxicity induced by 5-FU present with signs and symptoms of acute coronary syndromes with elevated cardiac biomarkers (troponin), and their ECGs often reveal ST segment changes. There can be two distinct clinical presentations, early or late presentation of cardiotoxicity. Early toxicity can occur during the infusion, whereas late presentation of toxicity can occur 1-2 days after the infusion. Usually, with early toxicity, troponin elevation may be evident. However, in the late presentation of cardiotoxicity symptoms, troponin elevation and/or ECG changes may be undetectable. Our case has a unique presentation of 5-FU toxicity in a patient developing ST elevation and non-sustained ventricular tachycardia as a late presentation of cardiotoxicity.

Despite the high magnitude of vasospasm with continuous infusion 5-FU administration (modified FOLFOX6 - infusional 5-FU, oxaliplatin, leucovorin), our patient was successfully treated and rechallenged with FLOX (bolus 5-FU, oxaliplatin, leucovorin) neoadjuvant chemotherapy. Because our patient manifested malignant ST elevation and VT during late presentation coronary spasm with 5-FU, the cardio-oncology multidisciplinary team administered a vasodilator during the pre-and post-treatment regimen. This regimen was described previously in the literature for late presentation of 5-FU cardiotoxicity.

This article was previously posted to the Research Square preprint server on August 15, 2022.

## Case presentation

**Figure 1:**
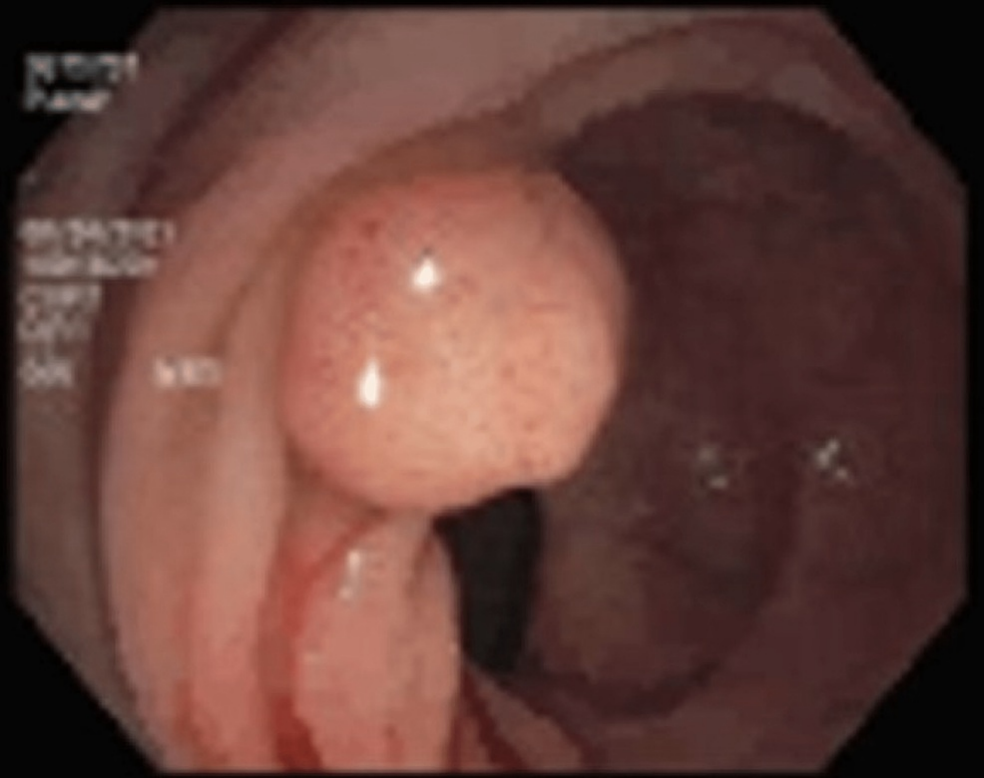
An ulcerated nonobstructing large mass was found 10 centimeters (cms) proximal to the anus. The mass was partially circumferential (involving one-half of the lumen circumference). Biopsies were taken with cold forceps for histology, and the area was tattooed with an injection of 3 mL of Spot (carbon black). The biopsies proved that the patient had rectal cancer.

A patient in his late 30s with no known significant past history or risk factors for cardiac disease was admitted to our hospital with chest pain. The patient was diagnosed with T3N0 rectal cancer in August 2021 (Figure 1).

He then received five sessions of radiotherapy treatment (XRT) and started chemotherapy approximately two months after his diagnosis. He had completed the 1st cycle of continuous infusion 5-FU delivered by his computerized ambulatory delivery device (CADD) pump. Approximately 18 hours into his scheduled 2nd cycle continuous infusion 5-FU, the patient started experiencing mid-sternal non-radiating chest pressure with an elevated heart rate and generalized weakness while he walked his dog. His chest pain was described by him as “minimal” whenever the patient walked his dog during the 1st infusion cycle. The pain was normally alleviated when he took two oral tablets of calcium carbonate (Tums) since he had a history of acid reflux. However, during the second cycle, the Tums failed to alleviate his discomfort. He began to develop worsening chest pain even with less exertion. He contacted our on-call oncology service who advised him that it could be reflux or coronary artery spasm and recommended that he be evaluated in the emergency room. He continued to have bouts of sternal chest discomfort, even during limited movement. After the patient was admitted and was in the emergency department, the pain recurred again. At the time, the patient had been receiving his second cycle of 5-FU CADD pump as a treatment for his rectal cancer. Due to the patient experiencing continued chest pain, the 5-FU pump infusion was discontinued, given its association with coronary spasm, angina, and myocardial infarction.

**Figure 2:**
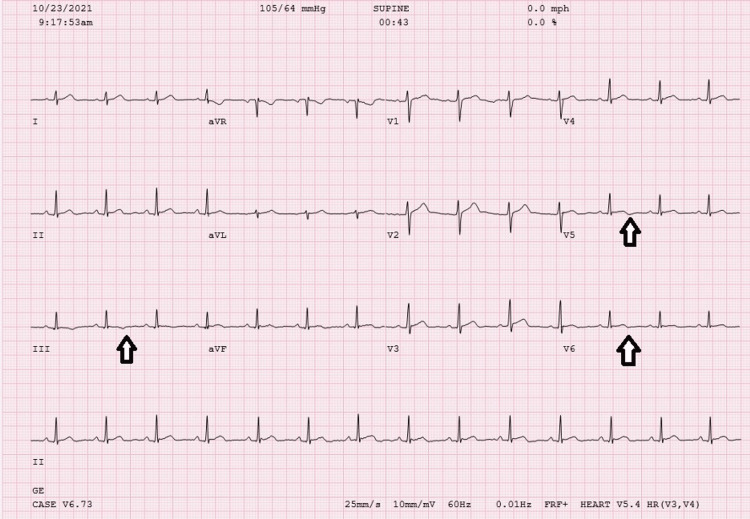
Baseline admission 12-lead ECG demonstrated sinus rhythm with mild nonspecific inferolateral ST T abnormality (arrows).

The computerized tomography (CT) chest angiogram did not reveal a pulmonary embolism. His admission ECG showed sinus rhythm with mild nonspecific inferolateral ST T abnormality (Figure 2). No significant ST elevation or depression was noted.

**Figure 3:**
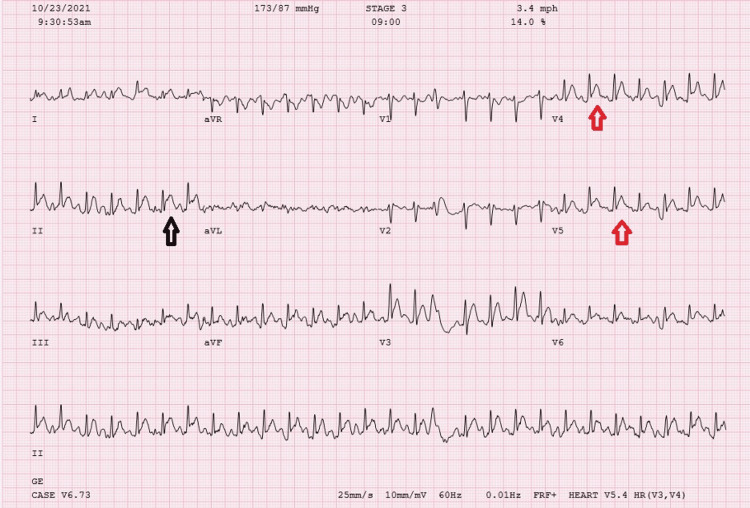
Peak stress 12-lead ECG, showing 2 mm ST elevation in the inferior leads (black arrow) and 1.5 mm ST elevation in V3-V6 leads (red arrows).

His initial troponin level was mildly elevated to 1.0 ng/ml, which subsequently decreased to 0.5 ng/ml (normal range: 0-0.04 ng/ml). Over the next 24 hours, he had no recurrence of chest pain or arrhythmia on the telemetry monitor. To evaluate the etiology of his chest pain, 26 hours after the 5FU pump was discontinued, the patient underwent an exercise treadmill stress test under the supervision of the cardiology team. Approximately 7-9 minutes into the exercise on the standard Bruce protocol, the patient started developing recurrence of chest pain with frequent premature ventricular contractions (PVCs) with 2 mm ST elevation in the inferior leads, and 1.5 mm ST elevation in V3-V6 leads (Figure 3).

**Figure 4:**
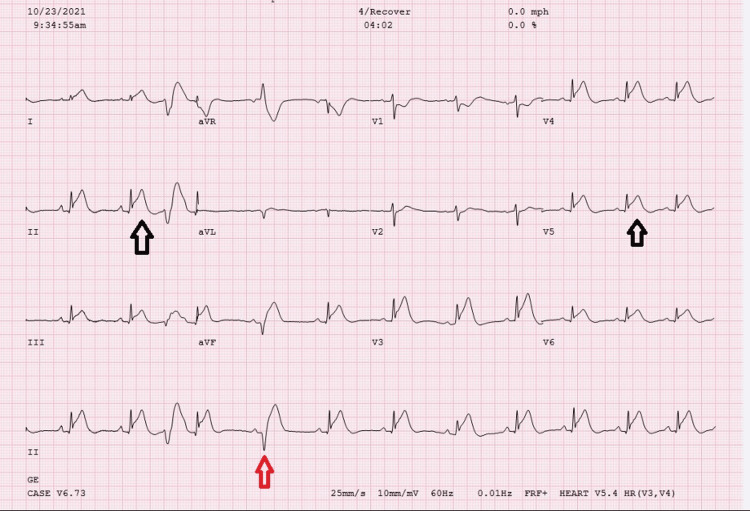
12-lead ECG 3 minutes into recovery 4/10 chest tightness HR 79 beats per minute (bpm), inferolateral ST elevation (black arrows) with PVC (red arrows). 12-lead ECG three minutes into recovery; 4/10 chest tightness, heart rate 79 beats per minute (bpm), inferolateral ST elevation (black arrows) with premature ventricular contractions (red arrows).

The stress test was terminated, and the ECG revealed extensive inferolateral ST elevation in the immediate recovery period with frequent PVCs, nonsustained ventricular tachycardia, and idio-ventricular rhythm. Sublingual (SL) nitroglycerine (NTG) was administered with resolution of the chest pain and inferolateral ST elevation (Figure 4).

The patient was taken for emergent cardiac catheterization, which revealed normal coronary arteries angiographically.

After discussing the plan of care with the patient's primary oncologist, the patient was admitted for the 3rd cycle of 5-FU via bolus (FLOX regimen) to the observation unit to reduce the tumor burden before his planned scheduled colorectal surgery.

Our patient was pretreated with nifedipine ER 30 mg five hours before, isosorbide mononitrate three hours before, and diltiazem 30 mg one hour before the planned administration of the 5-FU bolus. During bolus administration, the cardio-oncologist and the oncological pharmacist were at the bedside as the patient was monitored on continuous telemetry monitoring. A plan was established to immediately discontinue the 5-FU 12-minute bolus dose and to administer sublingual nitroglycerin (SL NTG) if chest pain recurs. The patient completed the scheduled 5-FU bolus administration without any complications or chest pain recurrence. Twelve hours later, the patient was discharged home. The patient was also re-administered nifedipine XL 30 mg the evening before and the morning after the 5-FU bolus administration. The patient successfully completed his planned neoadjuvant therapy as an outpatient without any recurrence of chest pain or repeat cardiac complications. This was accomplished via the three-drug pre-and post-treatment vasodilator regimen and 5-FU bolus administration (FLOX regimen).

## Discussion

5-FU is an antimetabolite drug that is used to treat cancer. It is usually administered to decelerate and prohibit cancer cell proliferation. It acts by inhibiting the enzyme thymidylate synthase by blocking the thymidine formation required for DNA synthesis [1]. The most common clinical manifestation of 5-FU cardiotoxicity is chest pain related to coronary vasospasm [2]. An increase in endothelin-1, a vasoconstrictor, and a decrease in prostacyclin, a vasodilator, is thought to be the cause of endothelial dysfunction, which may result in coronary vasospasm [3]. Patients experiencing cardiotoxicity induced by 5-FU present with signs and symptoms of acute coronary syndromes with elevated cardiac biomarkers (troponin), and their ECGs often reveal ST segment changes. There can be two distinct clinical presentations, early or late presentation of cardiotoxicity. Early toxicity can occur during the infusion, whereas late presentation of toxicity can occur 1-2 days after the infusion [4]. Usually, with early toxicity, troponin elevation may be evident. However, in the late presentation of cardiotoxicity symptoms, troponin elevation and/or ECG changes may be undetectable. Our patient developed ST elevation and non-sustained ventricular tachycardia as a late presentation of cardiotoxicity. Despite the malignant late presentation of this vasospasm with continuous infusion 5-FU administration (modified FOLFOX6), our patient was successfully treated and rechallenged with bolus 5-FU (FLOX) neoadjuvant chemotherapy. Because our patient manifested malignant ST elevation and ventricular tachycardia during late presentation coronary spasm with 5-FU, the cardio-oncology multidisciplinary team administered a vasodilator pre-and post-treatment regimen. This regimen was described previously in the literature for late presentation of 5-FU cardiotoxicity [5].

Lestuzzi et al., in a prospective study, evaluated the prevalence of exercise-induced myocardial ischemia in patients undergoing in-hospital long-lasting continuous infusion of 5-FU. The results showed a 10.3% global incidence of ischemia, as manifested by ECG changes, corresponding to previous observations reporting mostly ST-segment elevation, and less frequently, ST segment depression, negative T waves, nonspecific ST-T changes, QT interval prolongation, and arrhythmias [6].

Chakrabarti et al. performed a retrospective review of approximately ten patients to explore the safety of substituting FLOX for FOLFOX and CAPOX (capecitabine, oxaliplatin) in patients who had 5-FU-induced coronary vasospasm. Out of the 10 patients, eight patients had chest pain as the presenting complaint within 48 hours after beginning the 5-FU infusion. In nine out of the ten patients, coronary vasospasm occurred during the first cycle of therapy. All the patients made a full recovery after the discontinuation of infusion of 5-FU or capecitabine. Subsequently, all patients received FLOX from seven days to 18 months after the event, with seven patients treated within four weeks of the event. FLOX did not cause any cardiovascular adverse events in any of the 10 patients [7]. In a study conducted by Clasen et al., in patients with suspected fluoropyrimidine-induced coronary vasospasm, they successfully re-challenged them with 5-FU and completed their planned chemotherapy following cardioprotective pretreatment with two calcium channel blockers and a long-acting nitrate in conjunction with careful cardiac monitoring [8].

## Conclusions

Today, the survival of patients with advanced stages of colorectal cancer has improved. 5-FU is indicated for the treatment of patients with adenocarcinoma of the colon, rectum, stomach, breast, and pancreas. Although 5-FU was approved by the Food and Drug Administration (FDA) in 1962 (over 50 years ago) for the treatment of colorectal cancer, 5-FU is still utilized to treat patients with advanced stages of cancer and serves as the backbone therapy for colorectal cancer treatment. It is critical to educate patients about potential cardiac manifestations before treatment initiation and to identify early, at-risk (with known coronary artery disease or who develop any chest discomfort during or two-day post-infusion) patients for cardiac complications. This case report emphasizes the importance of increased awareness, vigilant monitoring, and a multidisciplinary cardio-oncology team’s involvement with high-risk cardiac patients receiving 5-FU-based chemotherapy. If the patient is experiencing symptoms of coronary vasospasm while undergoing 5-FU chemotherapy treatment, three-drug vasodilator therapy should be considered for administration before and after bolus 5-FU treatment during ongoing chemotherapy.
